# Resveratrol Inhibition of Rac1-Derived Reactive Oxygen Species by AMPK Decreases Blood Pressure in a Fructose-Induced Rat Model of Hypertension

**DOI:** 10.1038/srep25342

**Published:** 2016-05-03

**Authors:** Pei-Wen Cheng, Hui-Chieh Lee, Pei-Jung Lu, Hsin-Hung Chen, Chi-Cheng Lai, Gwo-Ching Sun, Tung-Chen Yeh, Michael Hsiao, Yu-Te Lin, Chun-Peng Liu, Ching-Jiunn Tseng

**Affiliations:** 1 Department of Medical Education and Research, Kaohsiung Veterans General Hospital, Kaohsiung, Taiwan; 2 Yuh-Ing Junior College of Health Care & Management, Kaohsiung, Taiwan; 3 Department of Diving Medicine, Zouying Branch of Kaohsiung Armed Forces General Hospital, Kaohsiung, Taiwan; 4 Institute of Clinical Medicine, National Cheng-Kung University, Tainan, Taiwan; 5 Institute of Clinical Medicine, National Yang-Ming University, Taipei, Taiwan; 6 Department of Internal Medicine, Division of Cardiology, Kaohsiung Veterans General Hospital, Kaohsiung, Taiwan; 7 Department of Anesthesiology, Kaohsiung Medical University Hospital, Kaohsiung Medical University, Kaohsiung, Taiwan; 8 Genomics Research Center, Academia Sinica, Taipei, Taiwan; 9 Section of Neurology, Kaohsiung Veterans General Hospital, Taiwan; 10 Department of Pharmacology, National Defense Medical Center, Taipei, Taiwan; 11 Department of Medical Research, China Medical University Hospital, China Medical University, Taichung 40402, Taiwan

## Abstract

Recent studies have reported that the activation of AMP-activated protein kinase (AMPK) suppressed oxidative stress. The aim of this study was to examine whether the activation of AMPK in the brain decreased Rac1-induced ROS generation, thereby reducing blood pressure (BP) in rats with fructose-induced hypertension. The inhibition of ROS by treatment with an AMPK activator (oral resveratrol, 10 mg/kg/day) for 1 week decreased the BP and increased the NO production in the rostral ventrolateral medulla (RVLM) of fructose-fed rats but not in control Wistar-Kyoto (WKY) rats. In addition, resveratrol treatment abolished the Rac1-induced increases in the activity of the NADPH oxidase subunits p22-phox and reduced the activity of SOD2, while treatment with an AMPK inhibitor (compound C, 40 μM/day) had the opposite effect, in the fructose-fed rats. Interestingly, the activation of AMPK abolished Rac1 activation and decreased BP by inducing the activities of extracellular signal-regulated kinases 1 and 2 (ERK1/2) and ribosomal protein S6 kinase (RSK) and nNOS phosphorylation in the fructose-fed rats. We conclude that the activation of AMPK decreased BP, abolished ROS generation, and enhanced ERK1/2-RSK-nNOS pathway activity by negatively regulating Racl-induced NADPH oxidase levels in the RVLM during oxidative stress–associated hypertension.

In late 2011, the United Nations declared that cardiovascular diseases associated with metabolic disorders, such as type 2 diabetes mellitus, obesity, and metabolic syndrome, had outpaced infectious diseases as the main global threat to human health^1^. The World Health Organization highlighted high fructose consumption, mainly in the form of sweetened beverages, as a risk factor for several metabolic diseases in humans^2^.

Nitric oxide (NO) is synthesized by the enzyme NO synthase (NOS). There are three different types of nitric oxide synthase: neuronal nitric oxide synthase (nNOS), endothelial nitric oxide synthase (eNOS), and inducible nitric oxide synthase (iNOS)^3^. NO inhibits the activation of the sympathetic nervous system (SNS) in the brain^4^. Our previous study revealed that ERK1/2-RSK signaling is involved in the regulation of nNOS to modulate blood BP in the NTS^5^. Previous studies have suggested that various environmental factors (salt consumption and/or obesity) affect the brain and cause sympathoexcitation via oxidative stress in the RVLM^6^,^7^. Fructose is widely known to cause oxidative stress and sympathetic overactivity^8^,^9^. The mechanisms underlying oxidative stress-induced sympathoexcitation in the brain that causes NO dysfunction during fructose-induced hypertension remain unclear.

The RVLM in the brain stem play important roles in cardiovascular regulation^10^,^11^,^12^. Angiotensin II (Ang II) increases the generation of reactive oxygen species (ROS) via NADPH oxidase during the RVLM neuron-mediated pressor response^12^,^13^. There is accumulating evidence that fructose promotes an oxidative imbalance by simultaneously enhancing ROS production and down-regulating key antioxidant enzymes such as SOD1 and SOD2^14^. Rac1 (small G protein) is a critical factor that increases NADPH oxidase expression and subsequently triggers ROS production^15^. Recent evidence has identified a role for Rac1 and Rac1-derived ROS in the NTS during cardiovascular regulation of hypertension *in vivo*; furthermore, inhibition of the Rac1-dependent ROS in the NTS decreases blood pressure in spontaneously hypertensive rats (SHRs)^15^ AMP-activated protein kinase (AMPK) is a serine/threonine protein kinase that serves as an energy sensor in the regulation of cellular metabolism. AMPK isoforms α1 and α2 consist of a catalytic α subunit and non-catalytic β and γ subunits^16^. AMPK is activated by changes in the AMP:ATP ratio that occur in response to energetic stress, and activation requires the phosphorylation of Thr172 in the activation loop of the catalytic α subunit^17^. Recent studies showed that the deletion of AMPKα2 enhanced the expression of NADPH oxidase subunits and induced oxidative stress in vascular endothelial cells^18^. AMPK activation was assessed based on the degree of T172 phosphorylation of the AMPK alpha subunit and S79 phosphorylation of acetyl-CoA carboxylase (ACC)^19^ Resveratrol is a potent activator of AMPK in neuronal cell lines, primary neurons, and the brain and has neuroprotective properties^20^. Resveratrol is known to promote antioxidant defenses by regulating a host of antioxidant enzymes^21^,^22^.

However, the effects of AMPK and Rac1-induced ROS in the brain on the regulation of fructose-induced hypertension *in vivo* are unknown. We hypothesized that the promotion of AMPK decreased Rac1-induced ROS generation, increased NOS and reduced BP during fructose-induced hypertension. Our results indicate that resveratrol decreased blood pressure during fructose-induced hypertension by activating AMPK, which in turn abolished the generation of ROS, induced SOD2 via lowering the activity of Rac1-induced NADPH oxidase, enhancing the activity of the ERK1/2-RSK-nNOS signaling pathway in the brain.

## Materials and Methods

### Reagents and Chemicals

All of the drugs, including urethane, fructose, resveratrol, compound C, and dimethyl sulfoxide (DMSO), and the mouse anti-actin, goat anti-rabbit, and goat anti-mouse IgG secondary antibodies were obtained from Sigma-Aldrich (Sigma Chemical Co., St. Louis, MO, USA). Anti-p-AMPK^T172^, anti-AMPK, anti-ACC^S79^, anti-p-ERK^T202/Y204^, anti-ERK, anti-nNOS^S1416^, anti-nNOS, anti-eNOS^S177^, anti-eNOS, anti-iNOS, anti-p-RSK^T359/S363^, and anti-RSK antibodies were purchased from Cell Signaling Technology (Beverly, MA, USA). Anti-p22-phox and anti-nitrotyrosine were purchased from Santa Cruz Biotechnology (Santa Cruz, CA, USA). Anti-p47-phox and anti-p67-phox were purchased from Millipore (Bedford, MA, USA). Anti-Cu/Zn-SOD and anti-Mn-SOD were obtained from StressGen Biotechnologies (La Jolla, CA, USA) and Abcam (Cambridge, UK), respectively.

### Animals

Sixteen-week-old male WKY rats were obtained from the National Science Council Animal Facility (Taipei, Taiwan) and housed in the animal room of Kaohsiung Veterans General Hospital (Kaohsiung, Taiwan). The rats were kept in individual cages in a light-controlled room (12-hour light/12-hour dark cycle), and the temperature was maintained between 23 °C and 24 °C. The rats were given normal rat chow (Purina; St. Louis, MO) and tap water ad libitum. All of the animal research protocols were approved by the Animal Research Committee. The institutional review board at Kaohsiung Veterans General Hospital approved all study procedures. The study was performed in accordance with approved guidelines. The study was carried out in compliance with the Helsinki Declaration.

The rats were acclimated to the housing conditions for 1 week. They were then habituated to the indirect blood pressure measurement procedure for 1 week. The rats were randomly assigned to four groups of four rats per group: 1) control group: WKY rats received an ICV injection of aCSF as a vehicle control; 2) fructose group: the WKY rats were provided with 10% fructose water for one week; 3) fructose + resveratrol group: the WKY rats were provided with 10% fructose water and oral resveratrol for one week; 4) fructose + resveratrol + compound C group: the WKY rats were provided with 10% fructose water and oral resveratrol and an ICV injection of compound C for one week. Ordinary tap water was provided to the control animals throughout the experimental period.

### ICV injection procedure

ICV infusion experiments were performed following a stabilization period of at least 30 minutes after insertion of the microinjector into the ventricular-guided cannula. The BP was monitored for 3 days after drug infusion. As a vehicle control, the effect of ICV injection of aCSF (142 mmol/L NaCl, 5 mmol/L KCl, 10 mmol/L glucose and 10 mmol/L HEPES, pH 7.4) was analyzed. intracerebroventricular (ICV) injection of compound C (40 μM/day) was initially dissolved in DMSO and then diluted in aCSF at a final concentration of 1% DMSO. The basal BP was examined prior to injection. The daily ICV drug infusions were performed over a 2-min period and delivered as a single bolus of a final volume of 5 μL from day 0 to day 7.

### Tissue Sample Collection

Animals were killed with an overdose of pentobarbital sodium (100 mg/kg, IP) and perfused intracardially with warm saline. The brain was rapidly removed and immediately frozen on dry ice. The medulla oblongata covering RVLM was blocked between 0.5 and 1.5 mm rostral to the obex, which was adopted from the atlas of Watson and Paxinos^23^ and served as the anatomical landmark (supplement Fig. 1). The ventrolateral or dorsomedial medulla, covering RVLM (approximately 1.5- to 2.5-mm lateral to the midline and medial to the spinal trigeminal tract), was collected by micropunches with a 1-mm inner diameter burr^24^,^25^. Tissues collected from the same experimental groups were pooled and stored at −80 °C.

### Blood pressure measurement

The systolic blood pressure (SBP) of the rats was measured before the start of the fructose or resveratrol and compound C treatments (week 0) using a tail-cuff monitor (Noninvasive Blood Pressure System, SINGA, Taipei, Taiwan). The rats were placed in the fixer for 30 min at a constant temperature of 34 °C. During the measurement, six individual readings were obtained in rapid sequence. The highest and lowest readings were discarded, and the average of the remaining 8 readings was used. The SBP of the rats was measured every day at the same time.

### Flow cytometry

The NO concentration detected method was following Baruch *et al.*, and Strijdom *et al.* ’s published paper in Journal of Molecular and Cellular Cardiology and The EMBO Journal^26^,^27^. RVLM incubated at 37 °C for 45 min in PBS (with Ca2+/Mg2+) containing 400 units/ml collagenase type IV (Worthington Biochemical Corporation), and then manually homogenized by pipettation. Samples were centrifuged, supernatants removed and cells resuspended in fresh. Samples were centrifuged, supernatants removed and cells resuspended in fresh, DAF-2 DA-free buffer (10 μM DAF-2 DA) incubated for 30 min at room temperature followed by immediate FACS analysis. Final count cell populations usually contained 10,000 cells. Fluorescence in these cells was produced by oxidation of DAF-2 DA to its highly green-fluorescent DAF triazol (DAF-2T) for, and signals were recorded on a frequency histogram by logarithmic amplifiers. Fluorescence data are expressed as mean fluorescence (percentage of control). A Becton Dickinson FACSCalibur® analyzer was used to quantify fluorescence (excitation wavelength: 488 nm and emission wavelength: 530 nm) at the single-cell level, and data were analyzed using Cellquest® version 3.3 (Becton Dickinson) software.

### Determination of NO in RVLM

The RVLM (20 mg) were deproteinized using a Microcon YM-30 (Millipore, Bedford, MA, USA). The total amount of NO in the samples was determined via a modified chemiluminescence-based procedure using the Sievers Nitric Oxide Analyzer (NOA 280i; Sievers Instruments, Boulder, CO, USA) purge system (Li *et al.*, 2005). The sample (10 mL) was injected into a reflux column containing 0.1 mol/L of VCl_3_ in 1 mol/L of HCl at 90 °C to reduce any nitrates and nitrites into NO. The NO was then combined with the O_3_ produced by the analyzer to form NO_2_. The emission resulting from the excited NO_2_ was detected by a photomultiplier tube and digitally recorded (mV). The values were then interpolated to a standard curve of concurrently determined NaNO_2_ concentrations. The measurements were recorded in triplicate for each sample. The measured NO levels were corrected for the RVLM of the studied rats.

### ROS production in the RVLM

The endogenous *in vivo* O_2_^−^ produced in the RVLM was determined by staining RVLM slices with dihydroethidium (DHE) (Invitrogen, Carlsbad, CA). The RVLM dissected out of the studied rats was placed in OCT compound (Shandon Cryomatrix; Thermo Electron Co., Pittsburgh, PA), flash-frozen in a methylbutane-chilled bath, and then placed in liquid nitrogen. Cryostat slices (10 μm) were stained in the dark for 20 min at 37 °C in a humidified 5% CO_2_ incubator with 1 μM DHE. The samples were analyzed using fluorescence microscopy and Zeiss LSM Image software (Carl Zeiss MicroImaging, Jena, Germany).

### NADPH Oxidase Activity

NADPH oxidase activity of protein samples from RVLM was measured using the lucigenin-derived chemiluminescence method^28^. The luminescence assay was performed in phosphate buffer, containing 1 mM EGTA, 150 mM sucrose and 5 μM lucigenin as the electron acceptor, and 100 μM NADPH as the substrate. After dark adaptation, background counts were recorded and a tissue homogenate (100 μg protein) was added. The chemiluminescence value was recorded at 30-s intervals over 10 min. Superoxide production was measured after the addition of NADPH (100 μM) into the incubation medium as a substrate in the absence and presence of a flavoprotein inhibitor of NADPH oxidase, diphenyleneiodonium (10 μM). To minimize interference by light, all measurements were conducted in the dark room with temperature maintained at 22–24 °C. Light emission was recorded by a Sirius Luminometer (Berthold, Germany). Protein concentrations were determined using a Bio-Rad protein assay kit (Bio-Rad Laboratories, Hercules, CA). Data are presented as relative light units/sec/mg protein.

### Superoxide Dismutase Activity

The activity of SOD in samples from RVLM was measured using a SOD assay kit (Calbiochem)^29^. This assay kit utilizes 5,6,6a,11b-tetrahydro-3,9,10- trihydroxybenso[c]fluorine as the substrate. This reagent undergoes alkaline autoxidation, which is accelerated by SOD, and yields a chromophore that absorbs maximally at 525 nm. The SOD activity was measured according to manufacturer’s instructions. Specific activity was expressed as units/sec/mg protein.

### Immunoblot analysis

The RVLM was dissected by micro punch (1-mm inner diameter) from a 1-mm-thick brain stem slice at the level of the obex under a microscope. Total protein extract was prepared by homogenizing the RVLM in lysis buffer with protease inhibitor cocktail and phosphatase inhibitor cocktail, followed by incubation for 1 hour at 4 °C. The protein extracts (20 g per sample, as assessed by BCA protein assay; Pierce) were subjected to 7.5% to 10% SDS-Tris glycine gel electrophoresis and transferred to a polyvinylidene difluoride membrane (GE Healthcare, Buckinghamshire, UK). The membrane was blocked with 5% nonfat milk in TBS/Tween-20 buffer (10 mmol/L Tris, 150 mmol/L NaCl, and 0.1% Tween-20, pH 7.4) and incubated with anti-p22-phox, anti-p47-phox, anti-p67-phox, anti-Cu/Zn-SOD, anti-Mn-SOD, anti-p-AMPK^T172^, anti-AMPK, anti-p-ACC^S79^, anti-p-ERK^T202/Y204^, anti-ERK, anti-p-RSK^T359/S363^, anti-RSK, anti-p-nNOS^S1416^ (Abcam, Cambridge, UK), anti-nNOS, anti-p-eNOS^S1177^, anti-nitrotyrosine or anti-iNOS antibodies at 1:1,000 in PBST with 5% BSA at 4 °C overnight. Peroxidase-conjugated anti-mouse or anti-rabbit secondary antibodies (1:5,000) were used. The specific bands were detected with an ECL-Plus detection kit (GE Healthcare) and exposed to film. The developed films were scanned using a photo scanner (4490, Epson, Long Beach, CA) and analyzed with NIH Image densitometry analysis software (National Institutes of Health, Bethesda, MD).

### Immunofluorescent staining analysis

The brain stem was incubated in a rabbit-anti-phospho-AMPK^T172^ antibody (1:100) or mouse-anti-nitrotyrosine (1:100). After washing with PBS, the sections were incubated with green-fluorescent Alexa Fluor 488 donkey anti-rabbit IgG (1:200; Invitrogen, Carlsbad, CA, USA) at 25 °C for 2 hours. The sections were analyzed using fluorescence microscopy and Zeiss LSM Image software (Carl Zeiss MicroImaging).

### Rac1 activation assay

Rac1 activity was measured using a Rac1 Activation ELISA Assay Kit (Cytoskeleton, Denver, CO, USA) according to the manufacturer’s instructions. The Rac1 G-LISA™ kit contains a Rac-GTP-binding protein immobilized in the wells of a 96-well plate. Active, GTP-bound Rac1 in cell lysates binds to these wells, whereas inactive GDP-bound Rac1 is removed during the washing steps. The bound active Rac1 is then detected with a luminescent Rac1-specific antibody. The degree of Rac1 activation is determined by comparing readings from activated cell lysates with readings from non-activated cell lysates; Rac1 inactivation in tissue culture is generally achieved via serum starvation. Following the addition of the chemiluminescent substrate, the signals can be measured using a luminometer. The total lysate loaded into each well was limited to 10–100 μg in 200 μl.

### Statistical analysis

One-way analysis of variance (ANOVA) with Scheffe’s post-hoc comparison was performed to compare groups. Differences with P values < 0.05 were considered significant. All of the data are expressed as the means ± SEM.

## Results

### The activation of AMPK induced a systemic vasodepressor effect and NO release is through eliminating ROS production in the RVLM of rats with fructose-induced hypertension

**Figure 1:**
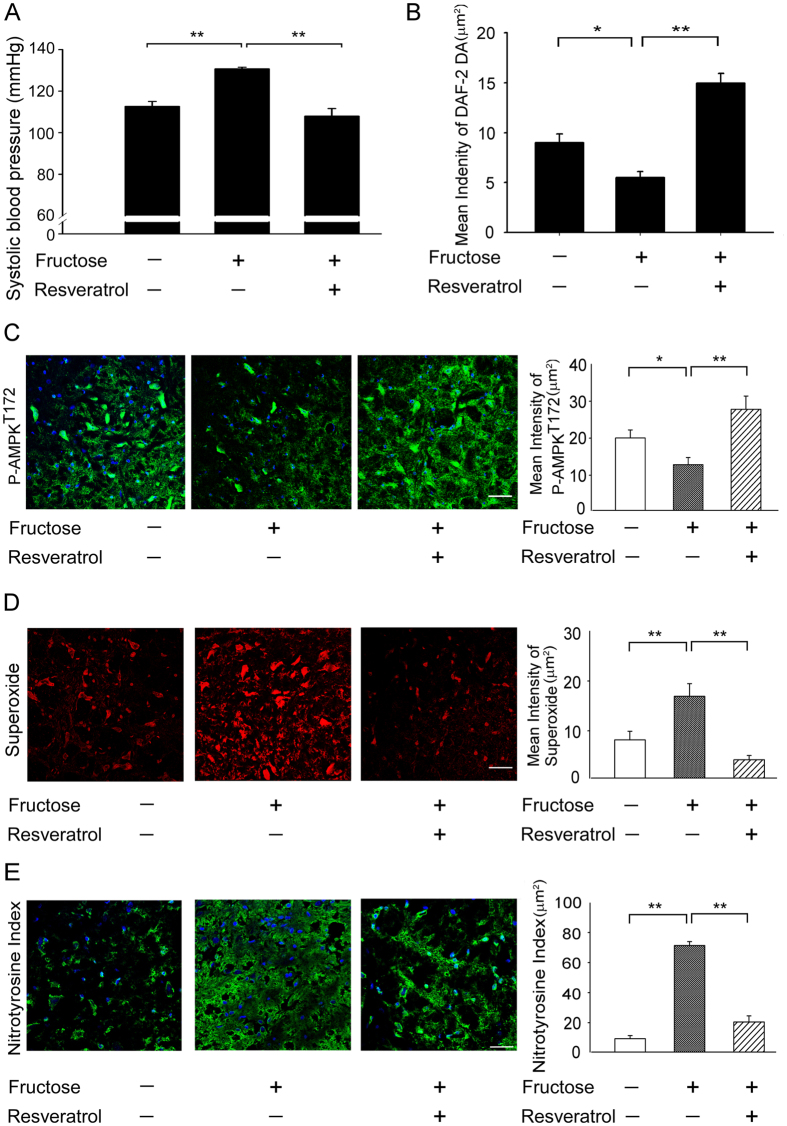
Resveratrol reversed the superoxide-dependent NO production and elevated SBP in the RVLM of rats with fructose-induced hypertension. (**A**) The graph reveals the effects of resveratrol on SBP in the fructose groups after one week. The SBP significantly recovered after the resveratrol treatment compared with the fructose group. (**B**) Quantitative analysis of intracellular NO, measured by flow cytometry of DAF-2 DA florescence intensity, in control, fructose and fructose + resveratrol (n = 10,000 cells per group; One-way ANOVA). Treatment with resveratrol significantly increased the NO levels in the RVLM in the fructose group compared with the control. (**C**) Confocal microscopy analysis of green fluorescence was used to estimate p-AMPKT172 levels in the RVLM after treatment with resveratrol. The representative images shown demonstrate that the elevation of the AMPK phosphorylation level was significantly increased in the RVLM after treatment with resveratrol. (**D**) Representative images of DHE-treated brain sections. Sections of the RVLM from the fructose group showed significant increases in DHE fluorescence compared with the control group sections. Furthermore, the DHE fluorescence in the RVLM were significantly attenuated by the resveratrol treatment. (**E**) Confocal microscopy analysis of green fluorescence was used to estimate nitrotyrosine levels in the RVLM after treatment with resveratrol. The representative images shown demonstrate that the elevation of the nitrotyrosine level was significantly decreased in the RVLM after treatment with resveratrol. The images were photographed at ×400 magnification. We examined 3 groups (control; 10% fructose-treated; and 10% fructose+resveratrol; n = 6 for each). The values shown are the means ± SEM, n = 6. *P < 0.05, **P < 0.01. Scale bar: 20 μm.

To investigate whether the ROS levels of fructose-fed rats were significantly higher in the RVLM, leading to down-regulation of NO release and inducing hypertension. We measured the nitrate levels and SBP in animals receiving both fructose and an AMPK activator (resveratrol). SBP was significantly higher and the NO levels in the RVLM were significantly decreased in the fructose-fed rats than in the control groups. (Fig. 1A,B, histogram 1 and 2). Interestingly, treatment with resveratrol markedly enhanced the NO levels in the RVLM and attenuated the SBP in the animals fed fructose and resveratrol (Fig. 1A,B, histogram 2 and 3).

Immunofluorescent staining also demonstrated that AMPKT172 phosphorylation was significantly attenuated in the animals that received fructose; however, treatment with resveratrol influenced AMPKT172 phosphorylation in the RVLM of fructose-fed rats (Fig. 1C). DHE fluorescence was used to estimate the superoxide levels in the RVLM of animals fed fructose for 1 week. Representative images are shown in Fig. 1D. The levels of DHE fluorescence in the RVLM sections were significantly higher in the fructose-fed group than in the control groups. Furthermore, the DHE fluorescence levels in the RVLM was significantly attenuated in the animals that received both fructose and resveratrol (Fig. 1D). Besides, immunofluorescent staining also demonstrated that nitrotyrosine was significantly enhanced in the animals that received fructose; however, treatment with resveratrol attenuated nitrotyrosine in the RVLM of fructose-fed rats (Fig. 1E). These results indicate that the elimination of ROS may be required for the AMPK-induced release of NO and the depressor response.

### AMPK activation impaired Rac1/NADPH oxidases and elevated SOD2 in the RVLM of rats with fructose-induced hypertension

To investigate whether resveratrol limits ROS production via inhibiting Rac1 activation of NADPH oxidases during fructose-induced hypertension, we examined the activity and expression of Rac1, NADPH oxidase subunits and SOD when both fructose and resveratrol were administered.

**Figure 2:**
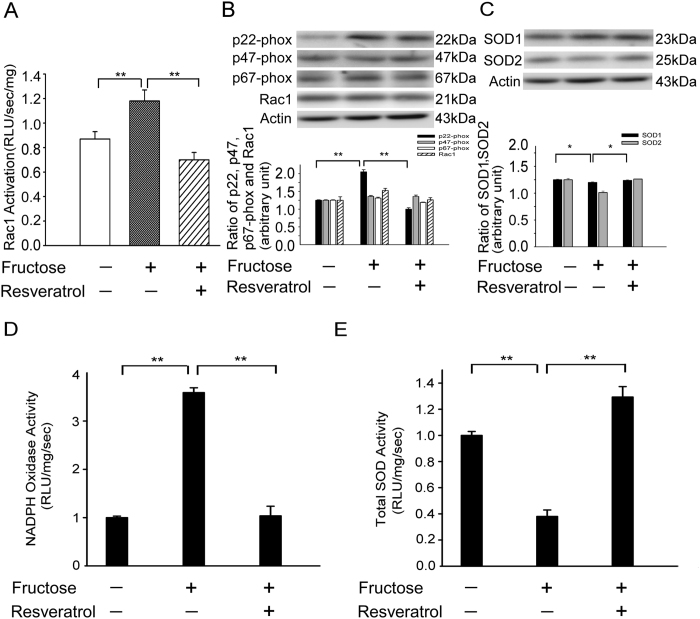
Resveratrol abolished the Rac1-induced increases in NADPH oxidase subunits (p67 and p22-phox) in the RVLM, respectively, and reduced the levels of SOD2 in the RVLM of rats with fructose-induced hypertension. (**A**) Bar graph showing the Rac1 activation ratio after treatment with fructose and/or resveratrol. Note the significant decrease in fructose-induced Rac1 activation after the administration of resveratrol. (**B**) Quantitative immunoblot analysis demonstrating that the NADPH oxidase subunit p22-phox ratio in the fructose group was reduced in the RVLM after treatment with resveratrol. (p22, p47, p67-phox, rac1 and actin the position of the 22, 47, 67, 21 and 43-kDa molecular weight marker is indicated, respectively). (**C**) Quantitative immunoblot analysis demonstrating that the level of SOD2 protein in the RVLM was significantly increased by the administration of resveratrol. (SOD1, SOD2 and actin the position of the 23, 25 and 43-kDa molecular weight marker is indicated, respectively). (**D**) Bar graph showing the NADPH oxidase activity ratio after treatment with fructose and/or resveratrol. Note the significant decrease in fructose-induced NADPH oxidase activity after the administration of resveratrol. (**E**) Bar graph showing the Total SOD activity ratio after treatment with fructose and/or resveratrol. Note the significant increase in fructose-reduced total SOD activity after the administration of resveratrol. The data shown represent the mean ± SEM of six independent experiments. *P < 0.05, **P < 0.01.

Rac1 activation in the RVLM was significantly enhanced in the fructose-fed group compared to the control group (Fig. 2A, histogram 1 and 2). Interestingly, the resveratrol treatment markedly inhibited Rac1 activation in the RVLM of the fructose-fed rats (Fig. 2A, histogram 2 and 3). We observed significantly higher relative p22-phox protein expression levels in the RVLM, respectively, of fructose-fed rats compared to the control groups; the co-administration of resveratrol prevented these increases (Fig. 2B). However, the relative expression levels of the SOD2 proteins was significantly lower in the RVLM, of fructose-fed rats compared to the control groups. However, the SOD2 expression levels remained within the range of the values recorded in the control when resveratrol was co-administered with fructose (Fig. 2C). NADPH oxidase activity and Total SOD activity also have similar result in the Fig. 2D,E.

These results indicate that the activation of AMPK abolished the Rac1-modulated increase in NADPH oxidase activity and decreases in SOD2 activities in the RVLM during fructose-induced hypertension.

### The activation of AMPK-induced NO release and the depressor response eliminate Rac1-induced NADPH oxidase in the RVLM of rats with fructose-induced hypertension

**Figure 3:**
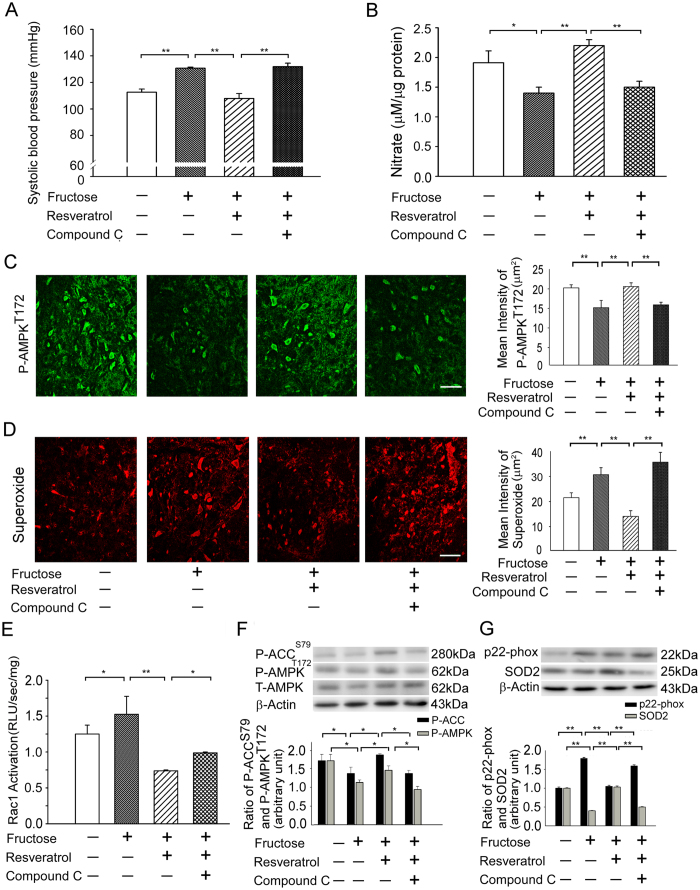
Activation of AMPK abolished the Rac1-induced increases in Rac1 and NADPH oxidase activities and reduced the activities of SOD2 in the RVLM of rats with fructose-induced hypertension. (**A**) Graph showing the effects of resveratrol on SBP with or without the administration of compound C. The SBP after treatment with resveratrol was significantly increased by compound C. (**B**) Levels of NO in the RVLM after the administration of compound C. The bar graph shows that the concentration of NO after treatment with resveratrol was significantly reduced by compound C. (**C**) Confocal microscopy analysis of green fluorescence was used to estimate p-AMPK^T172^ levels in the RVLM after treatment with resveratrol and compound (**C**). The representative images shown demonstrate that the elevation of the AMPK phosphorylation level in the RVLM after treatment with resveratrol was reduced by compound C. (**D**) Confocal microscopy analysis of DHE-treated brain sections in the RVLM after treatment with resveratrol and compound C. The representative images shown demonstrate that the elevation of the DHE fluorescence level in the RVLM after treatment with resveratrol was reduced by compound C. (**E**) Bar graph showing the activation ratio of Rac1 after treatment with resveratrol and compound C. The Rac1 activation in the RVLM after treatment with resveratrol was significantly inhibited by treatment with compound C. (**F**) Immunoblot showing P-ACC^S79^ and P-AMPK^T172^ protein levels after treatment with resveratrol and the AMPK inhibitor, compound C. The elevated ACC and AMPK phosphorylation levels in the RVLM after resveratrol treatment were reduced by treatment with compound C. (P-ACC^S79^, P-AMPK^T172^ and actin the position of the 280, 62 and 43-kDa molecular weight marker is indicated, respectively) (**G**) Immunoblot showing p22-phox protein levels after treatment with resveratrol and compound C. The resveratrol-reduced increase in the p22-phox activity ratios in the RVLM were further enhanced by compound C. However, the resveratrol-induced increase in the SOD2 activity in the RVLM was inhibited by compound C. (p22-phox, SOD2 and actin the position of the 22, 25 and 43-kDa molecular weight marker is indicated, respectively) The values are shown as the means ± SEM, n = 6. *P < 0.05, **P < 0.01. Scale bar: 20 μm.

To investigate whether abolish the activation of AMPK in the RVLM increased Rac1-induced ROS generation, thereby inducing blood pressure in rats with fructose-induced hypertension. In this study, we used an AMPK activator (oral resveratrol, 10 mg/kg/day) and inhibitor (compound C, 40 μM/kg/day) to demonstrate that AMPK is an important regulator of the Rac1-NADPH oxidase activity that regulates BP. Treatment with compound C restored the SBP in animals fed both fructose and resveratrol and abolished the resveratrol-induced increases in the NO levels in the RVLM (Fig. 3A,B). Immunofluorescent staining also demonstrated that treatment with resveratrol influenced AMPK^T172^ phosphorylation in the RVLM of fructose-fed rats; treatment with compound C attenuated resveratrol-induced AMPK^T172^ phosphorylation in the RVLM (Fig. 3C). Similarly, the DHE fluorescence or H2DCFCA levels in the RVLM was significantly attenuated by resveratrol; treatment with compound C abolished resveratrol-reduced ROS generation (Fig. 3D; supplement Fig. 2). Moreover, Rac1 activation in the RVLM was significantly attenuated by resveratrol; treatment with compound C abolished resveratrol-reduced Rac1 activation (Fig. 3E).

Immunoblot analyses of proteins extracted from the RVLM demonstrated that treatment with resveratrol increased ACC^S79^ and AMPK^T172^ phosphorylation in fructose-fed rats; the addition of an AMPK inhibitor (compound C) reversed the resveratrol-induced ACC^S79^ and AMPK^T172^ phosphorylation in the RVLM (Fig. 3F). Our studies showed that resveratrol abolished the Rac1-induced increases in the expression of NADPH oxidase subunits p22-phox and reduced the levels of SOD2 in the RVLM, but treatment with compound C reversed the resveratrol-induced effects on Rac1/NADPH oxidases and enhanced the expression of SOD2 in the RVLM (Fig. 3G). These results indicate that the AMPK pathway inhibited Rac1-induced NADPH oxidase expression, enhanced SOD2 expression, and induced the release of NO and the depressor response.

### The activation of AMPK enhanced the activity of the ERK1/2-RSK-nNOS pathway in the RVLM of rats with fructose-induced hypertension

**Figure 4:**
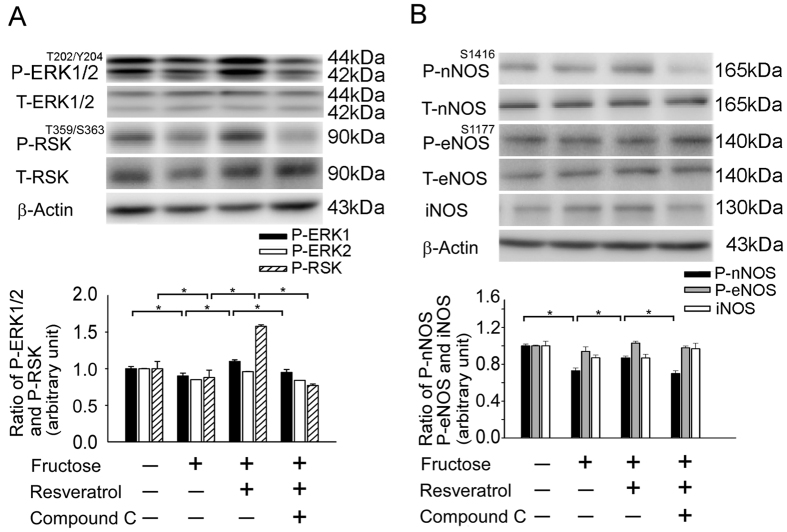
Activation of AMPK enhanced the activity of the ERK1/2-RSK-nNOS pathway in the RVLM of rats with fructose-induced hypertension. (**A**,**B**) Immunoblot showing the levels of P-ERK1/2^T202/Y204^, P-RSK^T359/S363^, P-eNOS^S1177^, P-nNOS^S1416^, and iNOS after treatment with resveratrol and compound C. (P-ERK1/2^T202/Y204^, P-RSK^T359/S363^, P-nNOS^S1416^, P-eNOS^S1177^, iNOS and actin the position of the 44/42, 90, 165, 140, 130 and 43-kDa molecular weight marker is indicated, respectively) The elevated ERK1/2, RSK, and nNOS phosphorylation ratios after treatment with resveratrol were reduced by compound C in the RVLM. The values shown are the means ± SEM, n = 6. *P < 0.05, **P < 0.01.

Our study demonstrated that AMPK-induced NO release and the depressor response may eliminate Rac1-induced NADPH oxidase. However, the mechanisms underlying the oxidative stress-induced NO dysfunction in the brain that causes sympathoexcitation during fructose-induced hypertension remain unclear. A previous study revealed that ERK1/2-RSK signaling was involved in the regulation of nNOS to modulate BP in the NTS^5^. Here, we used pharmacological approaches to further elucidate the involvement of the MEK-ERK1/2-RSK cascades in AMPK-induced nNOS phosphorylation. Immunoblot analyses of proteins extracted from the RVLM demonstrated that treatment with an AMPK activator (resveratrol) enhanced the phosphorylation of ERK1/2^T202/Y204^, RSK^T359/S363^, and nNOS^S1416^ in fructose-fed rats; the addition of an AMPK inhibitor (compound C) reversed these changes in the RVLM (Fig. 4). These results suggest that the MEK-ERK1/2-RSK pathways play a role in the AMPK-mediated depressor response in the RVLM.

## Discussion

**Figure 5:**
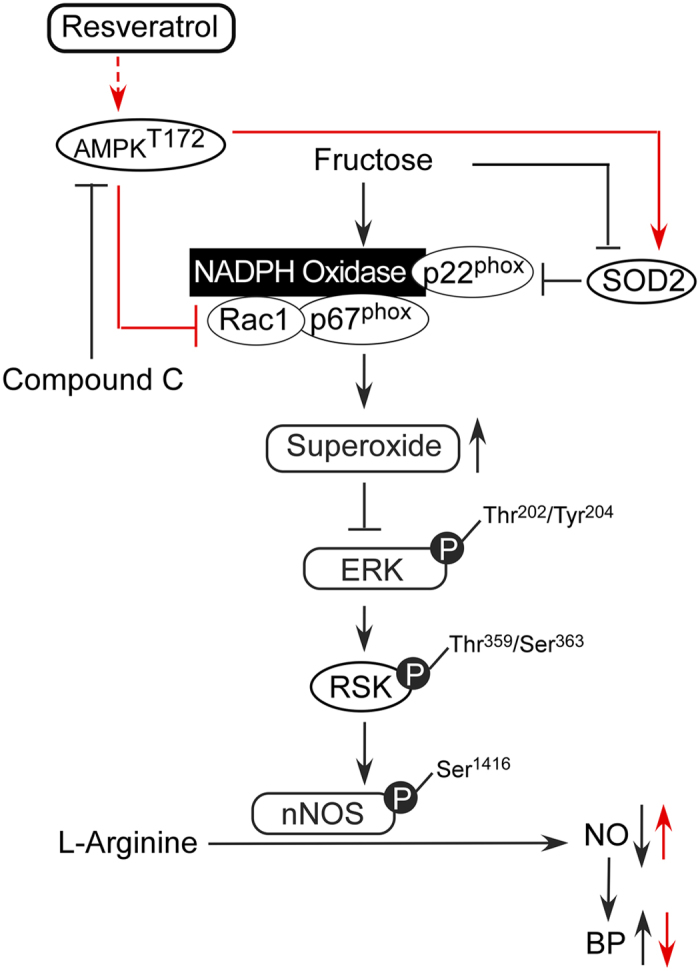
The proposed mechanism by which the AMPK signaling pathway regulates blood pressure (BP) in the RVLM of rats with fructose-induced hypertension. Treatments with an AMPK activator (resveratrol) and inhibitor (compound C) demonstrated that AMPK acts as an important regulator of BP through Rac1-NADPH oxidase activity. AMPK decreased blood pressure, abolished the generation of ROS and enhanced the activity of the ERK1/2-RSK-nNOS pathway by negatively regulating Racl-induced NADPH oxidase levels in the RVLM of rats with oxidative stress–induced hypertension.

The major findings of the present study are that the activation of AMPK decreased blood pressure, abolished the generation of ROS and enhanced the activity of the ERK1/2-RSK-nNOS pathway by negatively regulating Racl-induced NADPH oxidase levels in the RVLM during oxidative stress–induced hypertension (Fig. 5). Studies in yeast have clearly indicated that exposure to high fructose leads to increased levels of ROS, suggesting that the consumption of a high fructose diet (e.g., in the form of sweetened beverages) may be a risk factor for metabolic diseases in humans^1^,^30^. An imbalance of ROS production and elimination in the brain plays a pivotal role in the pathophysiology of a number of cardiovascular diseases associated with metabolic disorders, including hypertension, obesity, metabolic syndrome, type 2 diabetes, and dyslipidemia^31^,^32^,^33^. ROS in the brain are thought to contribute to the neuropathogenesis of hypertension by enhancing sympathetic nervous system activity. The key mechanism for reduced NO bioavailability is oxidative stress^34^,^35^. Oxidative stress can be defined as increased bioactivity of ROS relative to antioxidant defenses^36^. Oxidative stress reduces NO bioavailability via excessive production of superoxide, which reacts with NO to form peroxynitrite. Peroxynitrite in turn causes nitration of tyrosine residues on proteins (nitrotyrosine), providing a useful cellular marker of oxidative stress^37^. Recent studies have demonstrated that the ROS and nitrotyrosine levels in the RVLM were significantly higher in fructose-fed rats and that this elevation was associated with the down-regulation of NO release and the induction of hypertension (Fig. 1). Hyperglycemia, a consequence of diabetes, enhances the formation of advanced glycation end products (AGEs) and senescent protein derivatives that result from the auto-oxidation of glucose and fructose^38^. However, AGEs, along with their receptors (RAGEs), may directly induce the generation of ROS via NADPH oxidases and/or other previously characterized mechanisms^39^. In this study, we observed significant increases in the expression of NADPH oxidase subunits p22-phox in the RVLM of fructose-fed rats compared with control rats (Fig. 2). Fructose promotes an imbalanced oxidation state by simultaneously enhancing ROS production and down-regulating key antioxidant enzymes such as SOD1 and SOD2^14^. Recent studies also showed that the relative levels of SOD2 protein expression was significantly lower in the RVLM of fructose-fed rats than in control rats. However, the SOD2 expression levels remained within the range of values recorded in the control group when resveratrol was co-administered with fructose (Fig. 2C). Nozoe *et al.* reported that the transfection of SOD1, which scavenges ROS, into the NTS decreased blood pressure and HR^15^. Furthermore, the silencing of either AMPKα1 or AMPKα2 elevated oxidative stress by down-regulating genes involved in antioxidant defense, including SOD2, catalase, γ-glutamylcysteine synthase, and thioredoxin^40^,^41^,^42^. However, the opposite effect was observed after treatment with AMPK agonists such as metformin or AICAR, which inhibited hyperglycemia-induced intracellular and mitochondrial ROS production and increased the expression of peroxisome proliferator-activated response-γ coactivator-1α (PGC-1 α) and SOD2.

There is accumulating evidence that NADPH oxidase is the source of ROS in the brain. A previous study demonstrated that the inhibition of Rac1, which is a key regulator of NADPH oxidase, decreased sympathetic nerve activity in a rat model of hypertension^15^. Other recent studies showed that AMPKα2 deletion increased the expression of NADPH oxidase subunits and oxidative stress in vascular endothelial cells^18^. Therefore, we hypothesized that AMPK might be an important regulator of Rac1-NADPH oxidase activity. In this study, we used an AMPK activator (resveratrol) and inhibitor (compound C) to demonstrate that AMPK is an important regulator of the effect of Rac1-NADPH oxidase activity on BP (Fig. 3). Our study revealed a novel role for AMPK signaling in ROS homeostasis in the RVLM and the associated cardiometabolic phenotype.

NO is synthesized in cardiac myocytes and plays key roles in the modulation cardiovascular signaling. In response to H_2_O_2_, AMPK phosphorylates eNOS at serine 1177 but not nNOS in cardiac myocytes^43^ and phosphorylates eNOS at serine 633 in vascular endothelial cells^44^. Murphy *et al.*, used 7-NINA (the nNOS inhibitor) investigated that the effects of inhibition of nNOS and blocked to AMPK activation. Compound C reversed the increase in nNOS phosphorylation seen in the presence of AICAR. These founding demonstrated that AMPK and nNOS regulate the glucose sensitivity of ventromedial hypothalamic glucose-inhibited neurons^45^. Beside, Khan *et al.*, investigated that decreased activation of AMPK in nNOS KO mice indicate that AMPK activation is dependent on nNOS activity. AMPK activity has been also reported to phosphorylate Ser1412 of nNOS, thus increasing nNOS activity^46^. Our previous study revealed that ERK1/2-RSK signaling was involved in the regulation of nNOS in the NTS to modulate BP^5^. Moreover, Ang II may modulate central BP effects via ROS to down-regulate ERK1/2, ribosomal protein S6 kinase, and nNOS^11^. Current observations by showing that studies also fructose induced phosphorylation of IRS1^S307^ and reduced Akt^S473^ and nNOS phosphorylation^47^. However, we demonstrated that the activation of AMPK affected nNOS but not eNOS or iNOS during the depressor response mediated by the ERK1/2-RSK signaling pathway in the RVLM of rats with fructose-induced hypertension (Fig. 4). Thus, we propose that AMPK decreases blood pressure, abolishes the generation of ROS, and enhances the activity of the ERK1/2-RSK-nNOS pathway by negatively regulating Racl-induced NADPH oxidase levels in the RVLM during oxidative stress–associated hypertension (Fig. 5). There is accumulating evidence that NADPH oxidase is the source of ROS in the brain and has been identified as underlying pathogenic mechanisms associated with hypertension. In this study, our findings that the activation of AMPK induced a systemic vasodepressor effect and NO release may through eliminating ROS production in the RVLM of rats with fructose-induced hypertension. We demonstrated the importance of the activation of AMPK abolished the Rac1-modulated increase in NADPH oxidase activity and decreases in SOD2 activities in the RVLM during fructose-induced hypertension. We also investigated the cross talk between AMPK and Rac1 in the pathogenesis of hypertension. Our finding suggested that the activation of AMPK decreased blood pressure, abolished ROS generation, and enhanced ERK1/2-RSK-nNOS pathway activity by negatively regulating Racl-induced NADPH oxidase levels in the RVLM during oxidative stress–associated hypertension. However, the limitation of this study is due to use of pharmacological agent. Therefore, we will try to use gene silencing of AMPK in RVLM using lentivirus carrying small hairpin RNA inhibited AMPK expression in the future, which will help provide direct evidence of the permissive role of AMPK in RVLM in the molecular and blood pressure changes after fructose intake.

In conclusion, apart from its well-characterized activities as an antioxidant, accumulating evidence suggests that resveratrol eliminates the cardioprotective and chemopreventive actions of ROS. Resveratrol targets the central nervous system, can cross the blood–brain barrier, and induces neuroprotective effects^48^. The beneficial effects of resveratrol may be mediated through the activation of AMPK, which down-regulates factors such as Rac1, NADPH oxidases, and SOD2 and protects the central nervous system. These novel findings suggest that resveratrol, an AMPK activator, may be a potential pharmacological candidate for the treatment of hypertension.

## Additional Information

**How to cite this article**: Cheng, P.-W. *et al.* Resveratrol Inhibition of Rac1-Derived Reactive Oxygen Species by AMPK Decreases Blood Pressure in a Fructose-Induced Rat Model of Hypertension. *Sci. Rep.* **6**, 25342; doi: 10.1038/srep25342 (2016).

## Supplementary Material

Supplementary Information
