# Supplementary material for: Resveratrol Inhibition of Rac1-Derived Reactive Oxygen Species by AMPK Decreases Blood Pressure in a Fructose-Induced Rat Model of Hypertension
Source: Sci Rep. 2016 May 3;6:25342. doi: 10.1038/srep25342 (PMC4853785; doi:10.1038/srep25342)

Resveratrol Inhibition of Rac1-Derived Reactive Oxygen Species by AMPK Decreases Blood Pressure in a Fructose-Induced Rat Model of Hypertension

Pei-Wen Cheng, PhD[1,2]; Hui-Chieh Lee, MD[3]; Pei-Jung Lu, PhD[4]; Hsin-Hung Chen, MS[1,5]; Chi-Cheng Lai, MD[6]; Gwo- Ching Sun, MD, PhD[7]; Tung-Chen Yeh, MD, PhD[6]; Michael Hsiao, DVM, PhD[8]; Yu-Te Lin, MD, PhD[9]; Chun-Peng Liu, MD[6]; Ching-Jiunn Tseng, MD, PhD[1,5, 10,11]

[1]Department of Medical Education and Research, Kaohsiung Veterans General Hospital, Kaohsiung, Taiwan; [2]Yuh-Ing Junior College of Health Care & Management, Kaohsiung, Taiwan; [3]Department of Diving Medicine, Zouying Branch of Kaohsiung Armed Forces General Hospital Kaohsiung, Taiwan; [4]Institute of Clinical Medicine, National Cheng-Kung University, Tainan, Taiwan; [5]Institute of Clinical Medicine, National Yang-Ming University, Taipei, Taiwan; [6]Department of Internal Medicine, Division of Cardiology, Kaohsiung Veterans General Hospital, Kaohsiung, Taiwan; [7]Department of Anesthesiology, Kaohsiung Medical University Hospital, Kaohsiung Medical University, Kaohsiung, Taiwan; [8]Genomics Research Center, Academia Sinica, Taipei, Taiwan; [9]Section of Neurology, Kaohsiung Veterans General Hospital, Taiwan; [10]Department of Pharmacology, National Defense Medical Center, Taipei, Taiwan; [11]Department of Medical Research, China Medical University Hospital, China Medical University, Taichung 40402, Taiwan

Correspondence:
Ching-Jiunn Tseng, MD, PhD
Department of Medical Education and Research, Kaohsiung Veterans General Hospital, 386 Ta-Chung 1st Rd., Kaohsiung, Taiwan 813
E-mail: cjtseng@vghks.gov.tw
Tel: 886-7-3422121 ext. 1505; Fax: 886-7-3468056
or
Chun-Peng Liu, MD
Department of Administration, Kaohsiung Veterans General Hospital, Kaohsiung, Taiwan
E-mail:
cpliu@vghks.gov.tw
Telephone: 886-7-3422121ext. 2011

Pei-Wen Cheng and Hui-Chieh Lee contributed equally to this work.
Running title: AMPK Inactivates Fructose-Induced NADPH oxidase

Supplement Method

ROS production in the RVLM

The endogenous in vivo ROS produced in the RVLM was determined by staining RVLM slices with 2',7'-dichlorofluorescein (H2DCF) (Sigma Chemical Co., St. Louis, MO, USA). The RVLM dissected out of the studied rats was placed in OCT compound (Shandon Cryomatrix; Thermo Electron Co., Pittsburgh, PA), flash-frozen in a methylbutane-chilled bath, and then placed in liquid nitrogen. Cryostat slices (10 μm) were stained in the dark for 30 min at 37 ℃ in a humidified 5% CO_2 incubator with 10 μM H2DCF. The samples were analyzed using fluorescence microscopy and Zeiss LSM Image software (Carl Zeiss MicroImaging, Jena, Germany).

Supplement Figure

Supplement figure 1. Cross section of the medulla oblongata rostral to the obex, indicating the location of the RVLM.

Supplement figure 2. Resveratrol abolish the ROS generation in the RVLM of rats with fructose-induced hypertension. Confocal microscopy analysis of green fluorescence was used to estimate ROS levels in the RVLM after treatment with resveratrol. The representative images shown demonstrate that the elevation of the ROS level was significantly decreased in the RVLM after treatment with resveratrol. The images were photographed at ×400 magnification. We examined 4 groups (control; 10% fructose-treated; 10% fructose+resveratrol and 10% fructose+resveratrol+compound C; n=6 for each). The values shown are the means ± SEM, n=6. *P<0.05, **P<0.01. Scale bar: 20 μm.

Supplement figure 3. Full Western blots and fluorescent gel scans. Red boxes indicate regions cropped for main figure 2B-C.

Supplement figure 4. Full Western blots and fluorescent gel scans. Red boxes indicate regions cropped for main figure 3F-G.

Supplement figure 5. Full Western blots and fluorescent gel scans. Red boxes indicate regions cropped for main figure 4A-B.

Supplement figure 1.

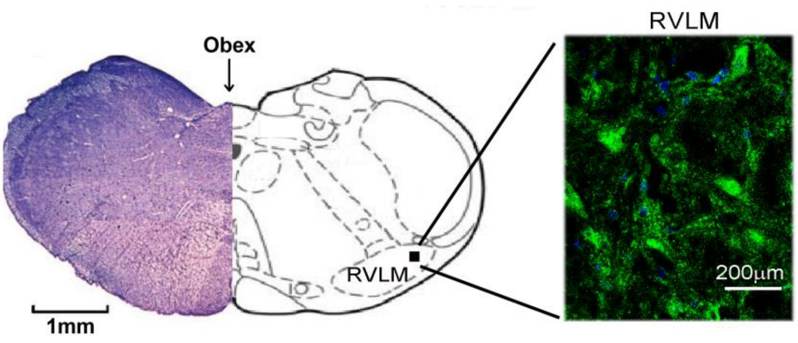

Supplement figure 2.

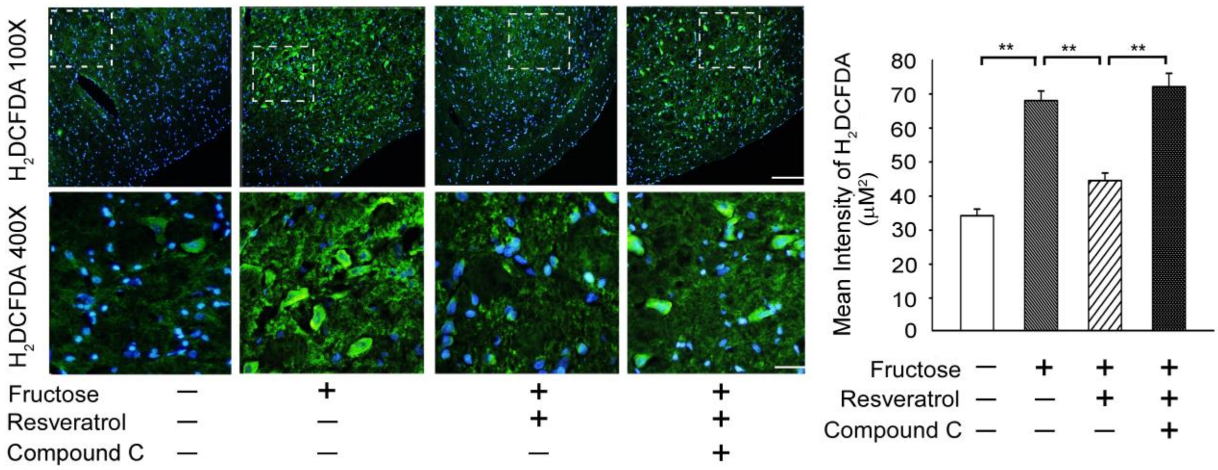

Supplement figure 3.

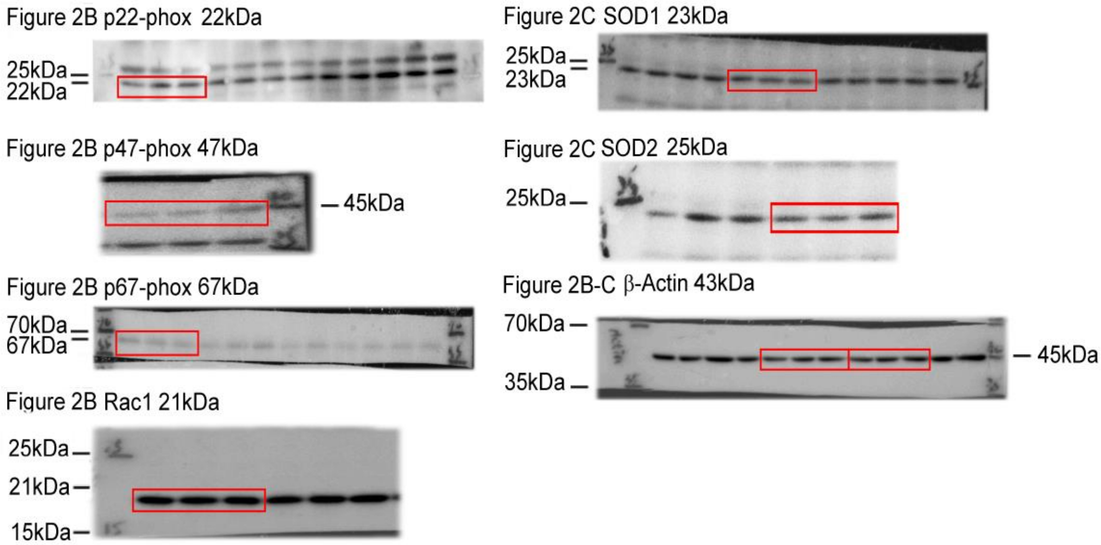

Supplement figure 4.

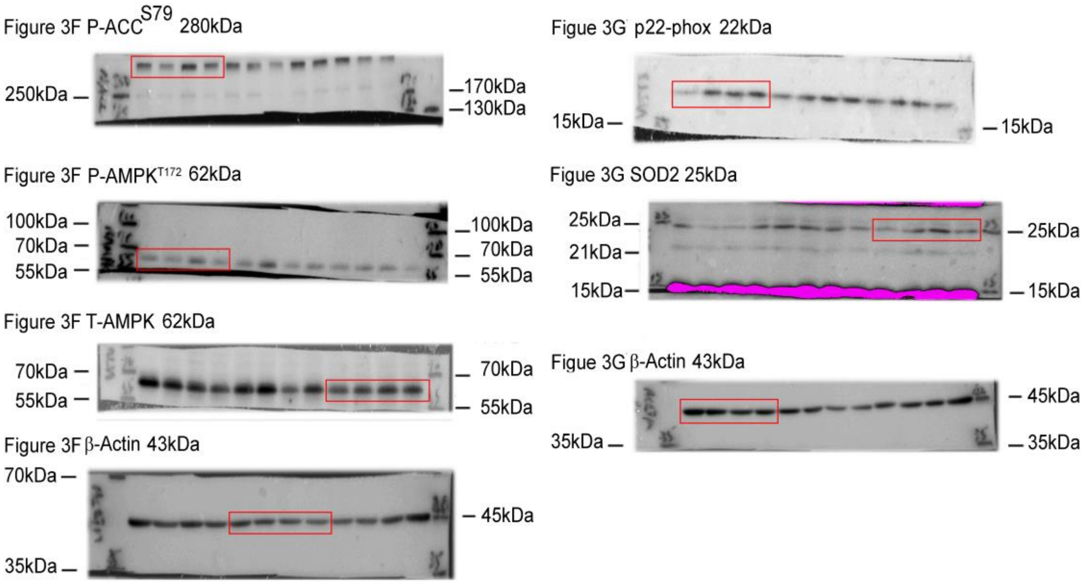

Supplement figure 5.

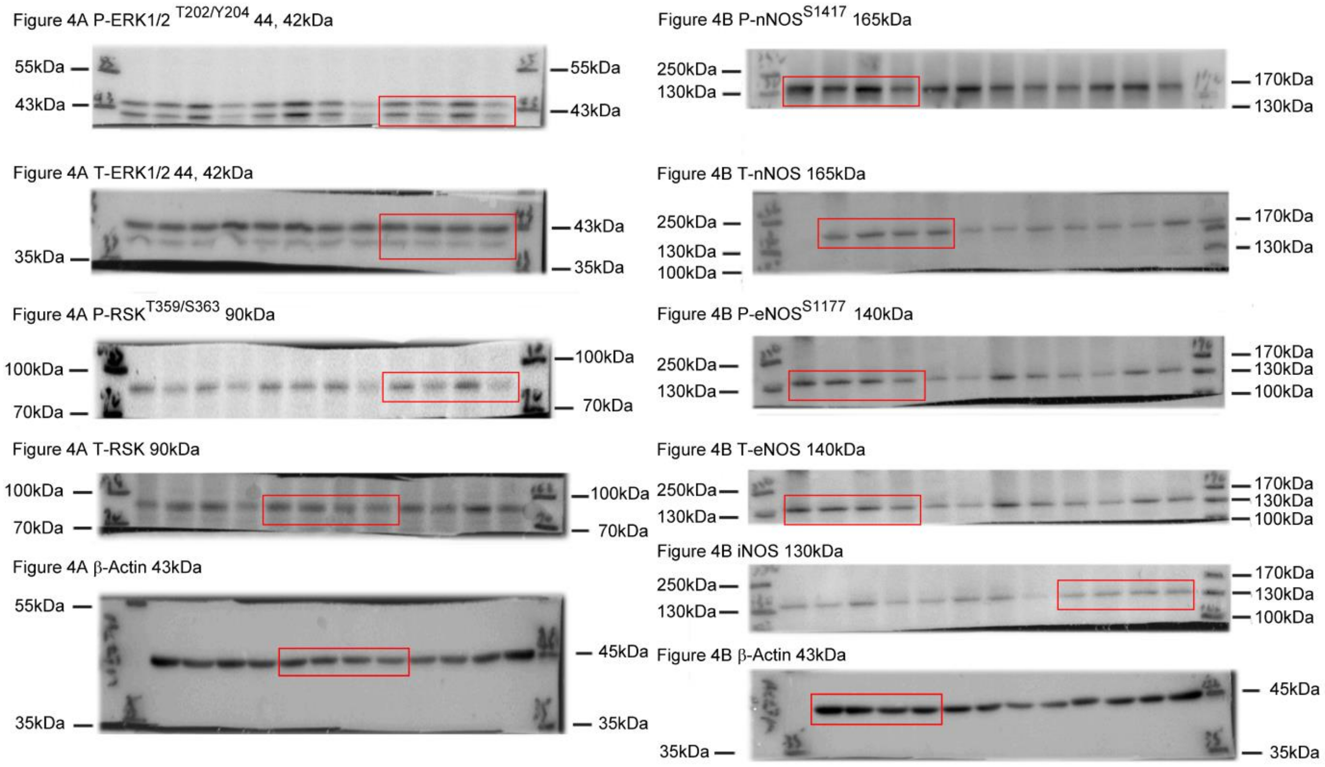

Supplement: Supplementary Information [file srep25342-s1.pdf]
